# Chlorhexidine as a Disinfectant in the Prosthodontic Practice: A Comprehensive Review

**DOI:** 10.7759/cureus.30566

**Published:** 2022-10-21

**Authors:** Hatem Alqarni, Ahmed Jamleh, Mark S Chamber

**Affiliations:** 1 Restorative and Prosthetic Dental Sciences Department, College of Dentistry, King Saud Bin Abdulaziz University for Health Sciences, Riyadh, SAU; 2 King Abdullah International Medical Research Center, Ministry of National Guard for Health Affairs, Riyadh, SAU; 3 Department of Head and Neck Surgery, Section of Oral Oncology and Maxillofacial Prosthodontics, The University of Texas, MD Anderson Cancer Center, Houston, USA

**Keywords:** acrylic, microbiome, microflora, microbiology, biofilm, impression disinfectants, disinfectant, antimicrobial, denture disinfectants, chlorhexidine

## Abstract

Introduction: Controlling the cross-contamination between the dental clinic and laboratory is of utmost importance to maintain the health of dental healthcare personnel (DHCP) and patients. The aim of this paper was to review the current literature with regard to the use of chlorhexidine as a prosthetic disinfectant in prosthodontic practice.

Materials and methods: A scoping review of the literature was performed in Medline/PubMed, Ovid Embase, and the Cochrane Library. A search for all literature published from 1980 to 2021 was based on the following keywords: [‘Chlorhexidine/gluconate’ OR ‘chlorhexidine', OR 'gluconate', OR 'denture disinfectants', OR 'antimicrobial', OR 'disinfectant', OR ‘impression disinfectants, OR prosthesis’ OR ‘biofilm, microbiology’] OR [teeth]. We reviewed the disinfectant in terms of its mechanism of action, antimicrobial effectiveness, disinfection techniques, clinical applications, corrosiveness/damage to the structure of prostheses, and reasonable shelf life.

Results: Chlorhexidine was tested under different concentrations ranging from 0.2 to 5%. It provided a significant reduction in biofilm viability but had a minimum effect on *Candida albicans* with a variable effect result that showed no significant differences in the dimensional changes by immersion of alginate dental impressions for no more than 10 minutes and no clinically significant dimensional differences on aluwax, polyether, condensation siloxane, and polyvinyl siloxane were noticed. Nonetheless, chlorhexidine altered the surface of the silicone and acrylic resins and affected the long-term hardness of the relining material.

Conclusion: Within the limitations of this review, the use of chlorhexidine disinfectant demonstrates a good measure in the reduction of contamination and cross-infection and has a minimal effect on the dimensional stability of most impression materials. Further studies with in-vitro testing are required to confirm these findings.

## Introduction and background

Pathogens' cross-contamination between dental clinics and laboratories is well documented and poses potential health risks to prosthodontic practice. The delivery of dental care involves dental healthcare personnel (DHCP) such as dentists, dental assistants, hygienists, and laboratory technicians. DHCP can be exposed to potentially pathogenic microorganisms such as hepatitis B virus, hepatitis C virus, Mycobacterium tuberculosis, staphylococci, streptococci, and other viruses and bacteria that colonize the oral cavity and upper respiratory tract [1]. Dental prostheses, at various stages of trial and insertion, may transmit infections from the patient to DHCP. Moreover, these prostheses are sometimes returned for repair or adjustments and exposed to oral microflora, including bacteria, fungi, and viruses. Therefore, DHCP working with these prostheses may be at a high risk of infection if proper disinfection procedures are not followed [2,3]. This may create a cycle of cross-contamination that may potentially expose other staff and patients to infections [4]. Therefore, the implementation of rigorous infection control practices at every stage is critical. According to the Centers for Disease Control and Prevention (CDC) guidelines, dental prostheses are considered semi-critical devices and must be subjected to high-level disinfection or sterilization [5]. Following a standard operating procedure for the disinfection of dental prostheses can minimize or even eliminate the spread of infections in the dental office and laboratory [5]. The disinfectant must have bactericidal, fungicidal, and virucidal properties. These include various formulations and combinations such as 5.25% sodium hypochlorite, 2% glutaraldehyde, 3% hydrogen peroxide, phenolic compounds, and 2-4% chlorhexidine gluconate (CHX). The U.S Food and Drug Administration (FDA) has issued recommendations for the use of FDA-approved disinfectants and high-level disinfectants for reprocessing reusable medical and dental devices [6]. Several studies have evaluated the effectiveness of various commercially available disinfection agents for dental prostheses by using microbial colony counts and scanning electron microscopy (SEM) that compared their effectiveness for different lengths of time, number of disinfection cycles, and techniques such as immersion, scrubbing, and spray disinfection [1,4-10]. Studies have also evaluated the effect of disinfectants on the physical and mechanical properties of denture base resins, such as flexural strength, surface hardness, and color [11-14]. Certain disinfectants, such as glutaraldehyde and alcohol-based substances have been found to alter the flexural strength and structure of the prosthesis, making it unsuitable for use [11,12].

Although many studies have been conducted to evaluate various prosthetic disinfecting agents and techniques, they lack rigorous evaluation criteria and standardization of evaluation. We conducted an extensive literature review to investigate CHX as the most commonly used prosthetic disinfectant. These results can be used to revise and develop infection control guidelines for dental practice.

## Review

Material and methods

A broad search of the literature on studies involving chlorhexidine gluconate was performed. A search of the dental literature was conducted in Medline/PubMed, Ovid Embase, and the Cochrane Library. The search for all articles published is based on the following keywords: Chlorhexidine/gluconate OR Chlorhexidine OR disinfectant agent/antiseptic OR clean OR cleaner OR cleanser OR disinfect OR antimicrobial OR anti-microbial OR antifungal OR anti-fungal OR microbiology OR microflora OR microbiome OR sanitize OR sanitizer OR acrylic acid resin/tooth prosthesis/exp denture/exp tooth crown/exp tooth implant/exp maxillofacial implant/exp maxillofacial obturator/palatal obturators/prosthodontics/tooth/abutment OR attachment OR alginate OR bite block OR bridge OR bridges OR cast OR coping OR crown OR crowns OR dental OR dentate OR denture OR device OR devices OR fluoride tray OR fixed partial OR framework OR guide OR housing OR hydrocolloid siloxane OR implant OR implants OR impression OR interim OR locator OR night guard OR obturator OR overdenture OR plastic insert OR poly-ether OR polyether OR polyvinyl OR poly-vinyl OR prosthesis OR prostheses OR prosthetic OR restoration OR acrylic resin OR reline material OR RPD OR screws OR splint OR splints OR stent OR stone OR surgical guide OR teeth or tooth, to review CHX’s mechanism of action, antimicrobial effectiveness, disinfection techniques, clinical applications, corrosiveness/damage to the structure of the prostheses, and shelf life. The studies were selected according to the following inclusion criteria: articles published in English from 1980 to 2021, in-vivo on human subjects and in-vitro studies; clinical trials and reviews involving the use of chlorohexidine gluconate as a prosthetic disinfection agent in dentistry. Articles published before 1980 or in languages other than English, studies involving animals, intraoral disinfection, case reports, non-randomized studies, and studies including confounding medications were all excluded.

Results

**Figure 1:**
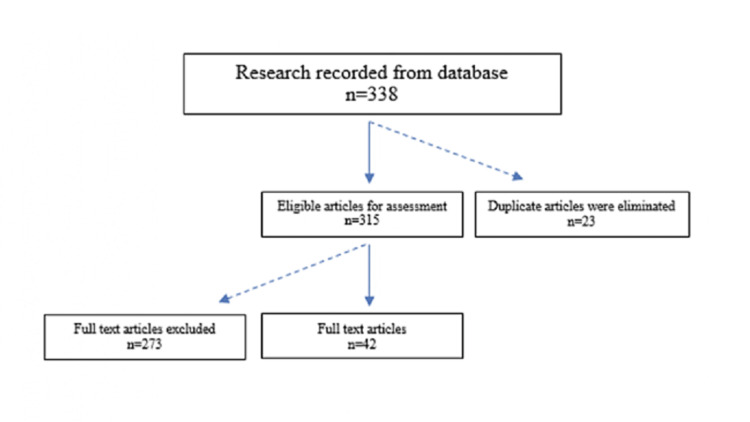
Flowchart of the selection of studies for the chlorhexidine gluconate comprehensive review

The first search, based on title/abstract analysis, returned a total of 338 potentially relevant articles. After careful review, 23 items were eliminated due to duplication. From the remaining 315 articles, another 273 were excluded due to exclusion criteria. Only 42 studies were considered eligible for full-text reading (Figure 1). This aspect was of interest in identifying differences in biofilms and dental materials.

Discussion

CHX is an antimicrobial substance belonging to the bisbiguanide class. It is a cationic bisbiguanide [1,6-di (4-chlorophenyl-diguanido) hexane] agent with a broad antibacterial spectrum (Gram-negative and Gram-positive) and some antiviral and antifungal properties [15]. It is also biocompatible with oral tissues and has the ability to remain on the surface and release gradually [15,16]. The most frequent oral preparation is water-soluble 0.12% CHX gluconate, which at physiological pH dissociates to release a positively charged molecule [17]. Its excellent properties have led to its increasing use in dentistry.

Mechanism of Action and Antimicrobial Effectiveness

CHX has a bactericidal effect, causing cell membrane rupture and consequent leak of intracellular fabric, including potassium (at low concentrations) or throw respiratory inhibition and nucleic acid loss (at high concentrations) [17]. CHX inhibits glycosyltransferase and a pair of phosphoenolpyruvate phosphotransferases, enzymes necessary for the function and maintenance of the bacterial glycolytic pathway [18].

In addition to yeast, CHX has a wide range of activity against Gram-positive and Gram-negative microorganisms. CHX is dependent on the type of microorganism. Gram-positive bacteria are more susceptible than Gram-negative bacteria. For instance, *Pseudomonas aeruginosa* (Gram-negative bacillus) [15], possibly due to the lack of the outer membrane and the presence of teichoic acid in the cell wall [17,18]. Furthermore, due to its adsorption to the oral surface, CHX exhibits a bacteriostatic effect, which can prolong the release time [19]. Furthermore, the 4% CHX solution did not demonstrate effectiveness against *Candida albicans*, where 50% of the candida were still alive after immersion for 10 minutes.

Disinfection Techniques

As evidenced by spore testing and chemical monitoring [20-22], the sterilization procedure is designed to destroy all micro­organisms, including resistant bacterial spores. The American Dental Association (ADA) has recommended that instruments that penetrate soft tissue or bone or come into contact with soft tissues be sterilized or discarded after each use [23]. This is generally accomplished by autoclaving at various temperatures and pressure levels for an acceptable amount of time with a specific item as stated by the manufacturer [21]. However, the existing impression materials cannot be heat sterilized. As a result, they are typically treated with an appropriate chemical disinfectant. This method is less effective than sterilization, which is categorized as an intermediate level of disinfection. Disinfectant agents must be effective in destroying microorganisms while having no negative impact on the dimensional stability or surface detail replication of impression materials. The ADA recommended disinfectants for use in dentistry such as phenol iodophors, quaternary ammonium compounds, sodium hypochlorite, glutaraldehyde, and CHX gluconate [20,22]. While surface disinfectants can eradicate the majority of harmful organisms, there is no method to test their efficacy. For example, immersion in a 2% glutaraldehyde solution for around 10 hours can eliminate bacterial spores and achieve sterilization, but this method of sterilization cannot be routinely verified by spore testing and is not recommended for items that can be sterilized with heat [20].

Many disinfection techniques, such as spray disinfection and immersion disinfection, have been proposed in the literature. Spray disinfection is more suited to materials that might otherwise undergo negative dimensional changes, such as alginate impressions. It is also simple to use and relatively inexpensive as just a small amount of disinfectant is dispensed. Immersion disinfection, on the other hand, is an excellent approach for increasing disinfectant exposure, particularly with chlorine dioxide. However, the latter is more expensive since a larger volume of disinfectant must be used compared to the spray method, especially if the material is unidosed and slightly more technique sensitive because an item must be entirely submerged [20].

Clinical Applications

Dental impressions: The use of dental impressions and resultant casts poured is a vital means of recording information in prosthodontics and general dental practice, as well as transferring this information from the chairside to the laboratory. Many different types of impression materials, with different physical and chemical properties, are in common use. Microorganisms can be transmitted through dental impressions and reproducible casts [23,24]. The ADA recommends that dental impressions should be rinsed to eliminate saliva, blood, and debris. It is recommended that impressions be rinsed under running water to remove any visible signs of contamination and disinfected with an appropriate agent before being poured or carried to the laboratory, and gloves must be worn when pouring impressions [25]. It is recommended that work be brought to the laboratory be received in a designated location, that impression be cleaned, disinfected and the personnel handling impressions wear protective clothes and disposable gloves [22]. A previous study surveyed dental practitioners in the United States on techniques for the disinfection of dental impressions. It was created to assess how successfully dental laboratory employees communicate with dentists on impression disinfection. Twenty-three percent of the laboratory directors were unaware of the disinfection procedure employed, and forty-seven percent were unaware of the time involved. Almost 50% reported receiving insufficient guidance on the disinfection techniques, and there was no set disinfection protocol to follow [26].

Disinfection of non-elastic impression materials: There is insufficient literature on the effect of CHX disinfection regimens on non-elastic imprint materials. Olsen et al. investigated the effect of immersion disinfection on the sharpness and dimensional stability of zinc oxide-eugenol impression material and discovered that one-hour immersion in any of the seven different disinfectants produced no clinically relevant alterations [27]. The differences in dimension change between the disinfected and control groups were deemed clinically insignificant, and there was no negative influence on surface detail reproduction.

Disinfection of reversible hydrocolloid impression materials: There is no literature describing the use of CHX to disinfect impressions generated using reversible hydrocolloid impression materials. However, Townsend et al. investigated the effects of other disinfectants, including iodophor, glutaraldehyde, and phenol, and discovered that a 10-minute immersion or spray approach with these agents had no influence on the accuracy of reversible hydrocolloid materials [28].

Disinfection of irreversible hydrocolloid, polyvinyl siloxane, and condensation impression materials: Irreversible hydrocolloid alginate impression materials are widely used in dental practice because they are simple to use, affordable, and patient-friendly. Alginate materials are usually supplied as a powder that must be mixed with room-temperature tap water. Under ideal conditions, alginate materials have been proven to be among the most accurate currently known materials, although they are also negatively damaged by disinfectant immersion [29]. Setcos et al. investigated the effects of 30-minute immersion disinfection on alginate [30]. When compared to CHX, immersion in glutaraldehyde resulted in statistically significant alterations in linear dimension values. Scanning electron microscopy (SEM) analysis revealed that immersion of the impressions significantly altered the crystalline structure of the dental casts. Bergman et al. investigated the impact of disinfection agents, including 2% glutaraldehyde and 0.5% CHX, on the dimensional stability and surface detail reproduction of four alginate materials [31]. Immersion for one hour resulted in unacceptable dimensional changes in all four impression materials, leading to the conclusion that alginate materials should not be immersed for this time period. Ivaniš et al. compared these results to those for the immersion addition of siloxane, polyether, and poly (vinyl siloxane) [32]. Casts obtained from the immersed polyether samples revealed significant linear dimensional change and are not suitable for disinfection by immersion in CHX gluconate, but the other two materials underwent 24-hour immersion with linear dimensional changes within a clinically acceptable range. In terms of dimensional stability, it appears that alginate responds less favorably when immersed than some other elastic impression materials; nonetheless, spray disinfection is less detrimental than immersion [30]. In most instances, the reproduction of surface details was found to be satisfactory and while disinfection by immersion did cause some dimensional change, it was felt that the degree was unlikely to affect the clinical performance of the material [30-31]. In general, the disinfectant role should ideally serve a dual purpose; it should be an effective antimicrobial agent while also having no negative impact on the dimensional accuracy and surface features of the impression material and the resulting gypsum cast [32,33]. Several methods of disinfection for alginate impression materials have been proposed. In clinical practice, the spray and immersion methods are the most commonly used. However, these conventional strategies have a number of drawbacks, including a loss of surface detail and dimensional inaccuracy of the impression. Due to the challenges in disinfecting alginate impression materials, another strategy utilizing self-disinfecting alginate impression materials was devised. According to studies, this technique displayed superior dimensional stability to spray and immersion techniques while also saving disinfection time [34,35]. Al-Nema investigated the dimension change and setting time after incorporating the disinfectant into the alginate material itself by mixing alginate with 0.05% iodine, 0.5% CHX, and 0.5% sodium hypochlorite solutions [36]. The results revealed that there is no statistically significant difference in the linear dimensional change. Among the other groups, sodium hypochlorite added to alginate as a disinfectant produced the greatest dimensional changes. The setting time differed significantly between the tested groups. It was found that treating alginate with sodium hypochlorite and CHX disinfecting agents increased the material setting time. However, 0.5% CHX gluconate was found to produce the least dimensional changes in all the impression materials, while 0.5% sodium hypochlorite produced the maximum changes. In conclusion, there were no significant differences in dimensional changes found between the control alginate and self-disinfecting alginate. However, there are significant differences in the setting time [36].

Disinfection of interocclusal records materials: Gounder and Vikas used spray and immersion to evaluate and compare the effects of 0.5% CHX, 1% sodium hypochlorite, and 2% glutaraldehyde on the linear dimensional stability of Jet Bites (addition silicone), Aluwax, and Ramitec (polyether) interocclusal recording materials for 30 and 60 minutes by using spray and immersion techniques [37]. When disinfected for 30 minutes using a spray or immersion technique, no significant variation in the linear dimensions was seen in any of the groups. When polyether was immersed in sodium hypochlorite for 60 minutes, its dimensional variation increased considerably. Addition silicone had the least dimensional change, followed by polyether and Aluwax. Restrictions in the disinfection method and time length should be used to preserve the dimensions and surface of the recording in addition to microbial elimination. Nonetheless, using the disinfectants either by spray or immersion technique, the dimensional change was <0.5%, which was not clinically significant [37].

Disinfection of soft relining materials: Pavan et al. investigated the hardness of four long-term soft denture liners after disinfection treatments with chemical solutions (2% glutaraldehyde, 5% sodium hypochlorite, and 5% CHX) and microwave energy [38]. Molloplast-B (heat-processed silicone soft relining material) produced the maximum hardness value regardless of the disinfection approach. Mucopren (a long-lasting soft relining material) had an intermediate hardness value, while Ufi Gel P and Eversoft had the lowest. The disinfection with glutaraldehyde yielded the maximum hardness value For Molloplast-B, the number of disinfections had no influence on the hardness values of the materials investigated, including disinfection techniques, and the use of two disinfection cycles had no effect on the hardness values for any of the materials [38].

Disinfection of maxillofacial prostheses: Chair-side disinfection of dental prostheses before laboratory procedures is the key to keeping microbial contamination out of the dental laboratory [20]. Acrylic resin and silicone are the most common materials utilized to create maxillofacial prostheses. Silicone is considered the material of choice due to its flexibility, patient comfort, and texture, which is similar to human skin [39]. Normal skin is rich in microorganisms, fungi, and bacteria, which naturally inhabit the skin surface. However, under conditions such as excessive heat and moisture, in patients with poorly controlled diabetes mellitus, or after prolonged administration of antibiotics, these microorganisms can cause endogenous infections and form biofilms. Inadequate hygiene of dental and maxillofacial prostheses increases the susceptibility of infection of surrounding tissues. Guiotti et al. conducted a study to evaluate and compare the antimicrobial activity of conventional disinfectant solutions, including 4% CHX for 10 minutes, and mechanical washing with water and soap was conducted [40]. All disinfection solutions provided a statistically significant reduction in biofilm viability. Photomicrographs demonstrated that 4% CHX affected the polymer surface. The hygiene of silicone polymer maxillofacial prostheses is a delicate procedure that, if handled improperly, can accelerate material deterioration [41]. The 4% CHX solution was not an effective disinfectant against *C. albicans*, with the persistence of microorganisms of approximately 50% after 10 minutes of immersion. According to this study, the surface becomes impregnated with 4% CHX, resulting in a modified surface leading to increased surface unevenness. Exposure to disinfection solutions on a regular basis may interfere with the qualities of silicone, resulting in changes in color, hardness, and tear strength [42]. The period of contact of the prosthetic material with the disinfectant should be managed so that the texture of the silicone surface does not change, which may also modify color stability and the appearance of the prosthesis [43].

Corrosiveness/Damage to the Structure of Prosthesis

The best disinfectant should meet the majority of the needs of the ideal agent without causing any changes to the prosthesis’s structure. The main advantages of CHX disinfectant are that it is non-corrosive and does not destroy plastics or rubber materials; however, due to its toxicity, it must be handled with caution. In dentistry, roughness on the surface of restorative and prosthetic materials severely interferes with their qualities, potentially reducing durability and increasing porosity. In a study conducted by da Silva et al., the authors proved the differences in the superficial roughness of the prostheses when disinfected with six different disinfectant solutions, and CHX disinfectants did not exhibit significant surface change with a slight reduction in the prosthesis’s surface roughness [44]. However, other investigations, however, have found that 2% to 4% CHX solutions have a deleterious impact on the hardness and roughness of acrylic resins [45,46]. For the silicone maxillofacial prosthesis, frequent exposure to disinfection solutions may interfere with the characteristics and properties of silicone, causing changes in color, hardness, and tear strength [42]. The period of contact of the prosthetic material with the disinfectant should be controlled so that the texture of the silicone surface does not change, which may also modify color stability and the appearance of the prosthesis [43]. Immersion in 4% CHX for 10 minutes revealed changes in the polymer surface [40], which may contribute to increased microbial adhesion.

Shelf and Useful Life

When choosing a disinfectant, several variables must be considered. One of them is shelf life, which is defined as the maximum amount of time an unused product can be stored before its efficacy is no longer guaranteed by the manufacturer. The second factor is the useful life, which is the maximum period of time that a disinfectant will remain effective. A study by Ahn et al. to determine a "safe use" period following opening containers of CHX was conducted to measure the antimicrobial effects of CHX (2-10 μg/ml) at sublethal concentrations on six strains of *Burkholderia cenocepacia* using chemical and microbiological assays. CHX (2, 4, and 10 μg/ml) kept at 23 °C for 42 days showed nearly the same concentration and toxicity compared with freshly prepared CHX on *B. cenocepacia* strains. When 5 μg/ml CHX and 20 μg/ml were spiked into six *B. cenocepacia* strains with varying inoculum sizes (10-10 CFU/ml), their toxic effects were not changed for 28 days. After 28 days of incubation, *B. cenocepacia* strains were detected in diluted CHX at concentrations up to 10 CFU/ml. Although abiotic and biotic changes in the toxicity of either antiseptic were seen, the results show that *B. cenocepacia* strains can survive in CHX for 28 days, emphasizing the significance of a control mechanism to monitor the likelihood of contamination in pharmaceutical products. To ensure public safety, unsealed drug items containing these preservatives or diluted antiseptics/disinfectants will require a safe-use period (shelf-life) warning [46,47].

Assessment of CHX's efficacy in eradicating several types of microbiome and investigation of its deleterious effects are not adequately elaborated. Therefore, further in-vitro/in-vivo studies are required. Table 1 and Table 2 shows the main characteristics of the included studies (type of material, first author, year of publication, results, disinfection procedures, type of chemical disinfectants, recommendations).

**Table 1:** Summary of recommended methods of disinfection using chlorhexidine

Effect	Disinfectant	Disinfection regimen	Results	Recommendation
Effect on impression materials	(A) Non-elastic impression material		There is no information in the literature on the effect of chlorohexidine disinfection on non-elastic impression material.	-
	(B) Reversible hydrocolloid impression material		There is no information in the literature on the effect of chlorohexidine disinfection on reversible hydrocolloid impression material.	-
	(C) Irreversible hydrocolloid impression material	Immersion	Immersion for 30 min has no effect [30].	Immersion is not recommended (more than 10 minutes)
			Immersion for 1 hour affects the dimensional stability and cast surface details, the recommendation should not be immersed for this time interval [31].	Immersion is not recommended (more than 10 minutes)
			Cast obtained from immersed alginate samples showed a greater percentage dimensional change compared to other impression samples [21].	Spray
			Disinfectant for 10 minutes causes no significant dimensional change in alginate impression and will produce cast with minimal dimensional change [21,33].	Spray
		Incorporated disinfectant within the alginate	Study by Al-Nema evaluated the dimension change and setting time by incorporating the disinfectant within the alginate material by mixing 0.5% iodine, 0.5% chlorhexidine, 0.5% hypochlorite solution, the result showed no significant difference in dimensional changes (chlorhexidine is the least dimensional change) but a significant difference among the test groups in setting time (chlorhexidine and sodium hypochlorite accelerated the setting time) [36].	-
	(D) Poly-vinyl siloxane	Immersion	Study by Ivaniš et al. Cast obtained from immersed poly-vinyl siloxane samples showed small linear dimensional alteration compared to other impression samples [32].	Immersion/spray
	(E) Condensation siloxane	Immersion	Study by Ivaniš et al. Cast obtained from immersed condensation siloxane samples showed small linear dimensional alteration compared to other impression samples [32].	Immersion/spray
	(F) Polyether	Immersion	Study by Ivaniš et al. Cast obtained from immersed polyether samples showed higher linear dimensional alteration compared to other impression samples [32].	Immersion is not recommended
Effect on interocclusal record	Polyether	Immersion/spray	Study by Gounder and Vikas evaluated the effect of different disinfectants and techniques (immersion or spray) for 30 and 60 minutes, polyether had significant dimensional variation when immersed in sodium hypochlorite for 60 minutes [37].	Immersion/spray
	Immersion/spray aluwax		The dimensional change using either spray or immersion technique did not have clinically significant [37]	
Effect on silicone materials		Immersion/spray	Study by Guiotti et al. evaluated the antimicrobial of 4% chlorhexidine for 10 minutes and mechanical washing with water and soap, all provided a significant reduction in biofilm, photomicrograph shows that 4% of chlorhexidine altered the surface of the polymer and was not effective against *C. albicans*, frequent exposure can change the color, hardness, tear strength and surface irregularity which contribute to increasing microbial adhesion [40].	Not recommended using chlorhexidine
Effect on relining denture materials	Molloplast-B	Immersion/spray	The highest value of hardness is for Molloplast-B using glutaraldehyde, independent of disinfectant technique.	Immersion/spray (chlorhexidine with precaution)
	Mucopren		The highest value of hardness is for Molloplast-B using glutaraldehyde, independent of disinfectant technique.	
	Ufi Gel		Eversoft has the lowest value of hardness [38].	
Corrosiveness/damage to the structure of prosthesis	Heal-polymerized cross-linked PMMA	Immersion/spray	Study by da Silva et al, the authors proved the differences in the superficial roughness in prostheses when disinfected with six different disinfectant solutions, and chlorhexidine disinfectants showed a not statistically significant change and slightly reduced in the surface roughness of the surfaces of the prostheses [44].	Not recommended using chlorhexidine
			Another study has also noted that 2% to 4% chlorhexidine solutions negatively affect the hardness and roughness of acrylic resins [45].	
		Immersion	Immersion of specimens in chlorhexidine and glutaraldehyde for 7 days produced a significant reduction in surface hardness [42].	Immersion/spray
			No significant effect on the surface hardness of the specimens was found after immersion in chlorhexidine solutions for 24 hours [13].	

**Table 2:** Type of chemical disinfectants and immersion recommended

Chemistry	Dilution	Exposure time
Chlorine compound 5.25% sodium hypochlorite	1:10	10 minutes [46,47]
Iodophors: Biocide	1:213	10 minutes [46,47]
Synthetic phenolic: OMC II	1:32	10 minutes [46,47]
Alcohol: Lysol spray	Full strength	3 minutes [20]
2% and 3.2% glutaraldehyde: ColsSpor	Varies	10 to 90 minutes [47]
Chlorhexidine gluconate: 0.12, 2, 4%	Full strength	10 minutes [46]

## Conclusions

The following conclusions were drawn based on this scoping evaluation of the literature: previous research examined the CHX at different concentrations, ranging from 0.2% to 5%. It is recommended that all impressions be disinfected with CHX by immersing them in as little as 0.2% concentration for a minimum of 10 minutes. The ability to perform this disinfection in the clinic is critical for keeping microbiologic contamination out of the dental laboratory, and the use of CHX as a disinfectant, either by spray or immersion technique, has been found to be effective in achieving good disinfection of dental impression materials and prostheses.
